# Blood Ammonia Level Correlates with Severity of Cirrhotic Portal Hypertensive Gastropathy

**DOI:** 10.1155/2018/9067583

**Published:** 2018-07-29

**Authors:** Ferial El-Kalla, Loai Mansour, Abdelrahman Kobtan, Asmaa Elzeftawy, Lobna Abo Ali, Sherief Abd-Elsalam, Sahar Elyamani, Mohamed Yousef, I. Amer, H. Mourad, Mohamed Elhendawy

**Affiliations:** ^1^Tropical Medicine and Infectious Diseases Department, Faculty of Medicine, Tanta University, Tanta, Egypt; ^2^Hepatology and Gastroenterology Department, Faculty of Medicine, Kafrelsheikh University, Kafr Elsheikh, Egypt; ^3^Clinical Pathology Department, Faculty of Medicine, Tanta University, Tanta, Egypt

## Abstract

**Background:**

Portal hypertensive gastropathy (PHG) is a common anomaly with potential for bleeding found in portal hypertension. Blood ammonia levels correlate well with liver disease severity and existence of portosystemic shunts. Increased ammonia results in vasodilation and hepatic stellate cell activation causing and exacerbating portal hypertension.

**Objective:**

To assess the relation of blood ammonia to the presence and severity of portal hypertensive gastropathy in cirrhosis.

**Methods:**

This cross-sectional study included 381 cirrhotics undergoing screening for esophageal varices (EV) divided into a portal hypertensive gastropathy group (203 patients with EV and PHG), esophageal varix group (41 patients with EV but no PHG), and control group (137 patients with no EV or PHG). A full clinical examination, routine laboratory tests, abdominal ultrasonography, child score calculation, and blood ammonia measurement were performed for all patients.

**Results:**

Blood ammonia, portal vein, splenic vein, and splenic longitudinal diameters were significantly higher and platelet counts lower in patients with EV and EV with PHG than controls. Patients having EV with PHG had significantly higher bilirubin and ammonia than those with EV but no PHG. Severe PHG was associated with significantly higher ammonia, EV grades, and superior location and a lower splenic longitudinal diameter than mild PHG. The PHG score showed a positive correlation with blood ammonia and a negative correlation with splenic longitudinal diameter.

**Conclusions:**

Blood ammonia levels correlate with the presence, severity, and score of portal hypertensive gastropathy in cirrhosis suggesting a causal relationship and encouraging trials of ammonia-lowering treatments for the management of severe PHG with a tendency to bleed.

## 1. Introduction

Portal hypertensive gastropathy (PHG) is a gastric mucosal anomaly commonly found in patients with portal hypertension [1]. It is more common and more aggressive in patients with cirrhosis than in those with portal hypertension due to noncirrhotic conditions [2].

The key element for the development of PHG is portal hypertension causing vascular congestion of the gastric mucosa with a resultant activation of cytokines, growth factors, and hormones that maintain and further the pathology which is characterized by capillary and venule dilatation, as well as congestion and tortuosity of the submucosal venules together with possible lamina propria stromal fibrosis and edema [3–5].

The endoscopic appearance is characterized by a mosaic or snake-skin-like appearance most commonly at the gastric body and fundus which may be associated with the presence of red spots [6].

PHG has been classified into mild and severe forms by different classification systems with the mild form in general having an appearance of mosaic-like pattern, scarlatiniform rash, superficial reddening, and snake-skin pattern, and the severe form displaying added red marks of variable diameter, with or without bleeding [7, 8]. The Baveno scoring system also classifies PHG into mild and severe using a score calculation that incorporates the presence or absence of gastric antral vascular ectasia (GAVE) [9]. This system has been found to be accurate in reflection of the bleeding risk from PHG among cirrhotic patients [10].

PHG is accountable for approximately 8% of nonvariceal upper gastrointestinal bleeding in hepatic patients [11]. Acute bleeding may infrequently complicate PHG especially when severe, yet chronic gastric mucosal bleeding leading to recurrent iron deficiency anemia is much more common and occurs in around 11–25% of cases [6, 7, 12, 13].

The major risk factors for PHG bleeding are increased duration, extent, and severity together with advanced cirrhosis, and eradication of esophageal varices by sclerotherapy or band ligation [3, 14].

Blood ammonia levels have been found to correlate well with the severity of liver disease and existence of portosystemic shunts, especially esophageal varices [15]. Accumulation of ammonia in splanchnic vessels in cases of liver function impairment results in vasodilatation and increased portal blood flow bringing about portal hypertension [16, 17]. There are indications that ammonia also affects endoplasmic reticulum dynamics and induces oxidative stress, both of which result in hepatic stellate cell (HSC) activation with production of multiple proinflammatory, contractile, and profibrogenic genes and proteins. A trial involving removal of ammonia in vitro, and lowering ammonia in vivo, has documented a downregulation of HSC activation markers with resulting reestablishment of HSC biology, suggesting that ammonia is a possible target for therapy directed at lowering portal hypertension [18].

PHG is a common manifestation of portal hypertension, and therefore, we aimed at finding out if blood ammonia levels correlate with the presence and severity of portal hypertensive gastropathy in cirrhotic patients.

## 2. Patients and Methods

This cross-sectional study was conducted on 381 cirrhotic patients positive for HCV or HBV attending the Tropical Medicine Endoscopy Unit, Tanta University, for endoscopic screening of esophageal varices. The study was conducted during the period from 7/1/2017 to 2/28/2018, 2018. The sample size of 381 was calculated based on a previous study by Kumar et al., who found the prevalence of portal hypertensive gastropathy in patients with cirrhosis to be 55%, with 5% precision and 95% confidence level [19]. Patients were excluded from the study if they had cardiac or kidney failure, hepatic encephalopathy or coma, active gastrointestinal bleeding, a history of bleeding during the previous two weeks to inclusion in the study, hepatocellular carcinoma, or portal vein thrombosis or were taking lactulose. Patients with factors affecting the portal pressure and gastric mucosal vascularity and blood flow such as those taking beta blockers and those for whom a previous intervention for varices (sclerotherapy, endoscopic band ligation, or scleroligation) or PHG (argon plasma coagulation or gastric mucosal band ligation) had been performed were also excluded.

A total of 381 patients were enrolled in the study. They were divided into three groups: (1) the portal hypertensive gastropathy group—203 patients with esophageal varices (EV) and PHG, (2) the esophageal varix group—41 patients with EV but no PHG, and (3) the control group—137 patients with no EV or PHG.

A detailed history was taken; a full clinical examination, routine laboratory tests, and pelvic-abdominal ultrasonography were performed for all participating patients after obtaining an informed written consent. Child-Pugh scores were calculated for each patient.

To overcome potential bias of results, study collaborators who were not involved in the upper endoscopy procedures screened and enrolled the participants, and all endoscopists were blinded to the study outcome.

### 2.1. Upper GI Endoscopy

Upper GI endoscopy was performed for all the patients. Classification of varices was performed according to the Japanese classification [20]. This involved recording of location (L), form (F), color (C), and red color signs (RC) of the varices. Classification and scoring of portal hypertensive gastropathy were performed according to the Baveno II scoring system; the mild mucosal mosaic pattern was given 1 point, and the severe pattern, 2 points. Isolated red markings were given 1 point, and confluent markings, 2. The presence of gastric antral vascular ectasia (GAVE) was noted and a further 2 points added to the score when flat or slightly raised, red stripes were seen radiating from the pylorus to the antrum and body of the stomach. PHG is considered mild when the score is ≤3 and severe at a score ≥ 4 [9].

### 2.2. Measurement of Blood Ammonia Level

A venous blood sample was collected from each patient after a minimum of 6 hours of fasting and 9 hours of abstinence from smoking. All patients had been instructed not to perform any physical exercise the day before.

The samples were taken at the laboratory, put in EDTA-containing sterile specimen tubes, and separated within 15 minutes of collection to be analyzed immediately. These precautions are necessary as the ammonia content of standing blood increases spontaneously due to generation and release of ammonia from red blood cells and due to deamination of amino acids by enzymes in the blood. Analysis was performed by the enzymatic method using reagents manufactured by BIOLABO SAS, Maizy, France.

### 2.3. Statistical Analysis

Statistical analysis was carried out using SPSS, version 15 for Microsoft Windows (SPSS, Chicago, Illinois, USA). The statistical data were reported as mean ± SD, frequencies (number), and percentages when appropriate. A comparison of numerical variables between the study groups was performed using one-way analysis of variance test followed by post hoc Tukey's multiple comparison test when data were normally distributed. Student's *t*-test was used to compare independent samples from two groups when the samples were normally distributed. Pearson's correlation was used to quantify the association between continuous variables. To compare categorical data, the *χ*^2^ test was performed. *P* values less than 0.05 (two-tailed) were considered statistically significant.

### 2.4. Ethical Considerations

All participating subjects provided written informed consents. The study protocol conforms to the ethical guidelines of the 1975 Declaration of Helsinki as reflected in a prior approval by the institution's human research committee. The study was approved by the Ethics Committee of the Faculty of Medicine, Tanta University (approval code 31588/06/17).

## 3. Results

**Figure 1:**
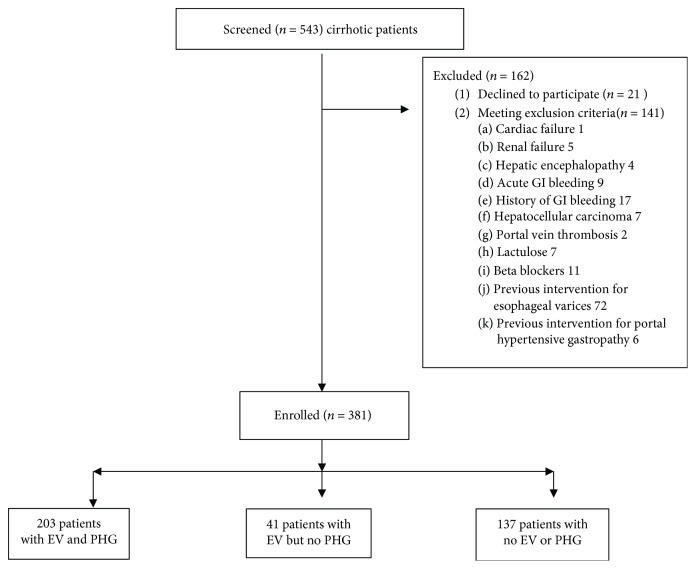
Study analysis population. *n*: number of patients; EV: esophageal varices; PHG: portal hypertensive gastropathy.

In total, 543 cirrhotic patients attending the Tropical Medicine Endoscopy Unit, Tanta University, were screened for participation in this study. 162 were excluded due to the presence of exclusion criteria in 141 patients and refusal to participate by 21. Thus, 381 cirrhotic patients were enrolled in this cross-sectional study; they were 258 males (67.7%) and 123 females (32.3%) (Figure 1). Testing for HCV RNA was positive for 377, whereas only 4 patients were HBV DNA positive. The participants were divided into three groups with no significant differences regarding distribution of age, sex, or Child-Pugh class: (1) the portal hypertensive gastropathy group—203 (53.28%) patients with EV and PHG, (2) the esophageal varices group—41 (10.76%) patients with EV but no PHG, and (3) the control group—137 (35.96%) patients with neither EV nor PHG (Table 1).

Patient baseline characteristics and demographic data are presented in Table 1. Platelet counts were significantly lower among patients with EV and PHG as well as those with EV only than in controls (*P* < 0.001). We found the group with EV and PHG to have significantly higher serum bilirubin levels than either controls or patients with EV only (*P* = 0.007) (Table 1).

Blood ammonia was significantly higher among patients with EV than controls and among patients with EV and PHG than among those with EV only as well as controls (*P* = 0.001) (Table 1).

The portal vein, splenic vein, and splenic longitudinal diameters were significantly higher in patients with EV and PHG as well as those with EV only than in controls (*P* < 0.001) (Table 1).

By using the Baveno II PHG scoring system to classify our 203 patients with PHG, we found that 149 (73.4%) had mild PHG (score ≤ 3) and 54 (26.6%) had severe PHG (score ≥ 4) (Table 2).

There was a significant difference between mild and severe PHG patients regarding the grading of the accompanying EV (*P* = 0.002), with a higher percentage of EV grades 1, 3, and 4 among the severe cases. Of interest was the absence of grade 4 EV among the patients with mild PHG (Table 2).

A significant difference in EV location was noticed between mild and severe PHG patients (*P* = 0.013), with severe cases having higher percentages of association with locus superior EV than their counterparts with mild PHG (14.82% versus 3.36%, resp.) (Table 2).

We compared between mild and severe cases of PHG with regard to multiple variables and found no difference between them in respect to patient age, blood count, liver function tests, Child-Pugh scores, or portal vein diameter. On the other hand, cases with severe PHG had significantly higher levels of blood ammonia (192.69 ± 59.87 versus 151.39 ± 54.37 *μ*g/dL) (*P* = 0.026) and significantly lower splenic longitudinal diameters (*P* = 0.001) (Table 3).

The PHG score itself was significantly higher with higher locations of the varices (*P* = 0.046) (Table 4).

We further studied the correlation between the PHG score and differential variables which revealed a significant positive correlation with blood ammonia and hemoglobin levels, and a significant negative correlation with the splenic longitudinal diameter (*P* < 0.05) (Table 5).

## 4. Discussion

Portal hypertensive gastropathy (PHG) is a common gastric mucosal anomaly in patients with cirrhosis and portal hypertension [1]. The condition carries a potential risk of acute and chronic gastrointestinal bleeding among these patients [13]. Evidence suggests that the key factor for the development of PHG is portal hypertension [3].

Increased blood ammonia in cases of liver function impairment is a pathogenetic factor for the production of portal hypertension and has been suggested as a possible target for portal hypertension lowering therapy [16–18]. In the light of this data, we aimed at finding out if blood ammonia levels correlate with severity of portal hypertensive gastropathy in cirrhotic patients.

Platelet counts were significantly lower among patients with EV and PHG as well as those with EV alone than in controls (*P* < 0.001). It has been found by other recent studies that low platelet counts are associated with the presence of varices in viral cirrhotic patients [21–23].

Patients with EV and PHG in our study were found to have significantly higher serum bilirubin levels than either controls or patients with EV only (*P* = 0.007) indicating that the presence of PHG is related to severity of the hepatic disease. This finding is similar to that of the HALT-C Trial in 2006, which recorded that bilirubin was significantly higher among PHG patients than those without [24]. However, contrary to the HALT-C Trial, we did not record any significant worsening in albumin or INR levels.

Several authors have found a correlation between the presence of PHG and Child-Pugh stage [2, 6, 25]. In this study, there were no significant differences in Child class between the cases with or without PHG. This is congruent with the results of Spina et al., Primignani et al., Merkel et al., and Bellis et al. [8, 13, 26, 27].

Blood ammonia was significantly higher among patients with EV than controls (*P* = 0.001). Other studies have found this relation between the presence of varices and elevated blood ammonia and even suggested a blood ammonia cutoff value for prediction of variceal presence [15, 28–30]. This can be explained by the role ammonia plays in production of portal hypertension and hence development of varices [18].

Blood ammonia levels were significantly higher among patients with EV and PHG (162.12 ± 58.17 *μ*g/dL) than among those with EV but no PHG as well as the cirrhotic controls (153.04 ± 59.41 and 103.23 ± 72.76 *μ*g/dL, resp.) (*P* = 0.001). It is known that PHG pathogenesis is associated with a hyperdynamic state brought about by portal hypertension in which there is an increased splanchnic and total gastric blood flow and most probably a decreased gastric mucosal blood flow [31]. Ammonia has been postulated to be part of a vicious circle in which elevation of ammonia results from the presence of portal hypertension and portosystemic collaterals and then leads to a further increase in portal hypertension [32]. It has also been suggested that PHG is associated with increased portal hypertension [24]. This could explain the higher levels of ammonia in cases with both PHG and EV and suggests a causal relationship.

The portal vein, splenic vein, and splenic longitudinal diameters were significantly higher in patients with EV and PHG as well as those with EV only than in controls (*P* < 0.001), which is expected as these are indicators of raised portal pressure; however, we found no difference in these parameters between patients having PHG and those without PHG in contrary to the HALT-C Trial [24]. Zardi et al. concluded their study in 2015 with there being no relation between splenic vein diameter and PHG [25].

By using the Baveno II PHG scoring system to classify our 203 patients with PHG, we found that 149 (73.4%) had mild PHG (score ≤ 3) and 54 (26.6%) had severe PHG (score ≥ 4). This more frequent pattern of mild PHG is consistent with the recorded prevalence range of mild PHG (29%–57%) and of severe PHG (9%–46%) among patients with portal hypertension [1].

There was no difference between mild or severe PHG regarding Child-Pugh class as also recorded by Abbasi et al. and Primignani et al. [13, 33]. Esophageal varix form, color, red color signs, or presence of risky bleeding signs were also not significantly different between the two PHG forms. This is the opposite to the findings of Taranto et al. who reported that PHG severity was closely related to the hemorrhagic risk of esophageal varices and Child-Pugh criteria [34].

A significant difference was recorded between mild and severe PHG patients regarding the grading of the accompanying EV (*P* = 0.002), with a higher percentage of large EV (grades 3 and 4) with the severe form of PHG than with the mild form (29.9% versus 16.78%). Of interest was the absence of grade 4 EV among the patients with mild PHG pointing to the relationship between severity of both PHG and portal hypertension and the similar mechanism for development of EV and PHG in response to increased portal pressure. Multiple previous studies have also reached this same interpretation which is supported by the strong correlation between severity of PHG and hepatic venous pressure gradient (HVPG) [13, 24, 26, 33, 35]. Kim et al. in their study on 331 cirrhotic patients noted that patients with severe PHG had significantly raised HVPG than those with either mild or no gastropathy [35].

Regarding the location of the EV (*P* = 0.013), severe PHG cases had higher percentages of association with locus superior EV than their counterparts with mild PHG (14.82% versus 3.36%, resp.) (*P* = 0.013), and the PHG score itself was significantly higher with higher locations of the varices (*P* = 0.046), a further indication of the relation with increased portal pressure.

Severe PHG can cause acute or chronic bleeding in cirrhotic patients, yet we found no significant decrease in hemoglobin levels among patients with severe PHG. The reason may be the exclusion of patients with active gastrointestinal bleeding or a history of bleeding during the previous two weeks to the study to eliminate the effect on blood ammonia levels.

Child-Pugh class and liver function tests were no different statistically in cases with severe PHG than in those with mild PHG indicating that worsening and progression of the condition is not affected by worsening of the liver condition. Primignani et al. also reported an unexpected low prevalence of severe gastropathy in patients with Child C class [13]. Taranto et al. in 1994 concluded that the severity of the mosaic-like appearance correlated with the severity of liver dysfunction yet made no such conclusion regarding the presence or appearance of the gastric mucosal red spots [34].

Cases with severe PHG had significantly higher levels of blood ammonia than those with mild PHG (192.69 ± 59.87 *μ*g/dL versus 151.39 ± 54.37 *μ*g/dL, resp.) (*P* = 0.026). We recorded a significant positive correlation between PHG scores and blood ammonia levels (*P* = 0.037). Increased ammonia in splanchnic vessels in cases of liver function impairment results in vasodilatation and increased portal blood flow bringing about portal hypertension [16, 17]. The increased levels may also cause such an increase in the gastric blood flow aggravating the vascular congestion characteristic of PHG and provoking cytokines, growth factors, and hormones to further worsen the condition. Ammonia is also known to affect endoplasmic reticulum dynamics and induce oxidative stress [18]. To our knowledge, no other studies have been performed to study the relationship between ammonia levels and severity of PHG; therefore, we could not compare our results with any others.

Significantly lower splenic longitudinal diameters were recorded among our patients with severe PHG than those with mild PHG (*P* = 0.001). This is in disagreement with the results recorded by Kim et al. whose patients with severe PHG had higher splenic diameters than those with mild PHG or no PHG [35].

A significantly negative correlation of the PHG score was found with the splenic longitudinal diameter (*P* = 0.006). This may be due to the effect of factors other than portal hypertension that maintain and further the pathology of PHG such as cytokines, growth factors, and hormones [3–5]. Even though the development of PHG is related to the presence of portal hypertension, there is generally no direct correlation with the level of the portal pressure [36].

Another study from Egypt by Nashaat et al. found that mean splenic bipolar diameter (MSBD) showed a significantly positive correlation with the presence of OV but not with PHG [37].

There is controversy regarding the correlation of splenic size with portal pressure as Nedredal et al. [38] state that spleen size does not correlate well with portal venous pressure in contrary to splenic stiffness which may be another explanation for our finding.

We recorded an unexpected significant positive correlation between PHG scores and serum hemoglobin levels; this may be related to the lower spleen size and therefore to lower phagocytic activity resulting in a decreased incidence of anemia among our patients with severe PHG having no present or recent related gastric mucosal bleeding.

The limitation of this study was the absence of a patient group with portal hypertensive gastropathy and no esophageal varices.

## 5. Conclusions

Blood ammonia levels correlate with the presence, severity, and score of portal hypertensive gastropathy in cirrhosis suggesting a causal relationship and encouraging trials of ammonia-lowering treatments for the management of severe PHG with a tendency to bleed.

## Figures and Tables

**Table 1:** Patient baseline characteristics.

Parameter	PHG (*n* = 203)	EV (*n* = 41)	Controls (*n* = 137)	*P* value
Age, years (mean, SD)	53.57 ± 6.61	55 ± 8.72	52.33 ± 11.06	0.923
Sex: male, *n* (%)	142 (69.95)	31 (75.61)	85 (62.04)	0.456
Etiology of cirrhosis, *n* (%)				
Hepatitis C virus	199 (98.03)	41 (100)	137 (100)	0.471
Hepatitis B virus	4 (1.97)	0 (0)	0(0)	
Ascites, *n* (%)	90 (44.33)	17 (41.46)	39 (28.47)	0.063
History of hepatic encephalopathy, *n* (%)	28 (13.79)	4 (9.76)	6 (4.38)	0.089
Hemoglobin (g/dL)	11.15 ± 1.81	10.99 ± 1.52	11.31 ± 2.53	0.726
WBCs (×10^9^/L)	5.23 ± 2.22	5.15 ± 2.09	5.93 ± 2.60	0.180
Platelets (×10^9^/L)	101.70 ± 31.34^a^	100 ± 30.61^c^	125.54 ± 36.25	<0.001^∗^
ALT (U/L)	43.34 ± 17.49	35.54 ± 15.69	39.28 ± 23.34	0.129
AST (U/L)	62.20 ± 26.62	53.34 ± 30.71	56.64 ± 29.67	0.308
Serum albumin (g/dL)	3.03 ± 0.56	3.05 ± 0.42	3.24 ± 0.61	0.086
Total serum bilirubin (mg/dL)	1.83 ± 1.24^a,b^	1.29 ± 0.55	1.31 ± 0.92	0.007^∗^
International normalized ratio	1.29 ± 0.24	1.22 ± 0.19	1.24 ± 0.30	0.449
Creatinine (mg/dL)	0.97 ± 0.21	0.94 ± 0.27	0.96 ± 0.24	0.781
Blood ammonia (*μ*g/dL)	162.12 ± 58.17^a,b^	153.04 ± 59.41^c^	103.23 ± 72.76	<0.001^∗^
Child-Pugh class, *n* (%)				
Class A	97 (47.79)	22 (53.66)	77 (56.20)	0.067
Class B	66 (32.51)	17 (41.46)	44 (32.12)	
Class C	40 (19.70)	2 (4.88)	16 (11.68)	
Portal vein diameter (mm)	13.64 ± 1.81^a^	13.42 ± 1.49^c^	11.75 ± 2.11	<0.001^∗^
Splenic longitudinal diameter (cm)	15.84 ± 1.69^a^	15.75 ± 1.74^c^	14.28 ± 2.65	<0.001^∗^
Splenic vein diameter (mm)	11.19 ± 1.20^a^	11.10 ± 0.92^c^	8.55 ± 1.91	<0.001^∗^
Esophageal varices, *n* (%)	203 (100)	41 (100)	—	—

^∗^Statistically significant at *P* < 0.05; ^a^*P* < 0.05 when compared with controls; ^b^*P* < 0.05 when compared with esophageal varices; ^c^*P* < 0.05 when compared with controls. ALT: alanine aminotransferase; AST: aspartate aminotransferase: WBCs, white blood cells; EV: esophageal varices; PHG: portal hypertensive gastropathy.

**Table 2:** Relationship of PHG severity with demographic, clinical, and endoscopic characteristics.

Parameter	Portal hypertensive gastropathy severity (*n* = 203)				*P* value
	Mild (*n* = 149)		Severe (*n* = 54)		
	*n*	(%)	*n*	(%)	
Sex					
Male	105	70.47	37	68.52	0.965
Female	44	29.53	17	31.48	
Child-Pugh class					
Class A	76	51.01	21	38.89	0.539
Class B	41	27.52	25	46.29	
Class C	32	21.47	8	14.82	
EV grading					
Grade 1	56	37.58	25	46.29	0.002^∗^
Grade 2	68	45.64	13	24.07	
Grade 3	25	16.78	12	22.22	
Grade 4	0	0.00	4	7.41	
EV red color sign (RC)					
RC0 (none)	84	56.38	38	70.37	0.063
HCS (hematocystic spots)	20	13.42	0	0.00	
CRS (cherry red spots)	8	5.37	4	7.41	
RWM (red wale markings)	37	24.83	12	22.22	
EV form (F)					
F1	84	56.38	29	53.70	0.973
F2	44	29.53	16	29.63	
F3	21	14.09	9	16.67	
Risk signs					
No	93	62.42	38	70.37	0.578
Yes	56	37.58	16	29.63	
EV location (L)					
LI (inferior)	96	64.43	25	46.29	0.013^∗^
LM (medialis)	48	32.22	21	38.89	
LS (superior)	5	3.36	8	14.82	
EV color (C)					
Cw (white varices)	101	67.78	41	75.93	0.535
Cb (blue varices)	48	32.22	13	24.07	
PHG mosaic pattern					
No	20	13.42	0	0.00	<0.001^∗^
Mild	121	81.21	16	29.63	
Severe	8	5.37	38	70.37	
PHG red markings					
No	121	81.21	8	14.82	<0.001^∗^
Isolated	28	18.79	13	24.07	
Confluent	0	0.00	33	61.11	
GAVE					
No	133	89.26	46	85.19	0.729
Yes	16	10.74	8	14.81	

^∗^Statistically significant at *P* < 0.05. EV: esophageal varices; PHG: portal hypertensive gastropathy; GAVE: gastric antral vascular ectasia.

**Table 3:** Relationship of portal hypertensive gastropathy severity with different variables.

Parameter	Portal hypertensive gastropathy severity (*n* = 203)		*P* value
	Mild (*n* = 149)	Severe (*n* = 54)	
	Mean ± SD	Mean ± SD	
Age, years	53.43 ± 7.71	58.00 ± 8.15	0.076
Hemoglobin (g/dL)	10.86 ± 1.62	11.97 ± 2.11	0.055
Platelets (×10^9^/L)	103.68 ± 32.44	96.08 ± 28.44	0.458
WBCs (×10^9^/L)	5.05 ± 2.22	5.74 ± 2.23	0.341
AST (U/L)	63.59 ± 29.48	58.23 ± 16.26	0.538
ALT (U/L)	43.16 ± 17.54	43.85 ± 18.03	0.905
Serum albumin (g/dL)	3.06 ± 0.59	2.95 ± 0.52	0.578
Total serum bilirubin (mg/dL)	1.92 ± 1.35	1.59 ± 0.89	0.413
International normalized ratio	1.33 ± 0.26	1.18 ± 0.17	0.056
Creatinine (mg/dL)	0.98 ± 0.20	0.95 ± 0.26	0.672
Child-Pugh score	7.19 ± 2.17	7.31 ± 1.93	0.863
Blood ammonia (*μ*g/dL)	151.39 ± 54.37	192.69 ± 59.87	0.026^∗^
Portal vein diameter (mm)	13.85 ± 1.68	13.07 ± 2.11	0.184
Splenic longitudinal diameter (cm)	16.31 ± 1.59	14.51 ± 1.26	0.001^∗^
Splenic vein diameter (mm)	11.39 ± 1.08	10.64 ± 1.39	0.052

^∗^Statistically significant at *P* < 0.05. ALT: alanine aminotransferase; AST: aspartate aminotransferase; WBCs: white blood cells.

**Table 4:** Relationship of portal hypertensive gastropathy score with demographic, clinical, and endoscopic characteristics.

Parameter	Portal hypertensive gastropathy score (*n* = 203)		*P* value
	*n*	Mean ± SD	
Sex			
Male	142	1.89 ± 1.05	0.954
Female	61	1.87 ± 1.13	
Hepatitis C virus			
No	4	1.00 ± 0.83	0.408
Yes	199	1.89 ± 1.07	
Hepatitis B virus			
No	199	1.89 ± 1.08	0.611
Yes	4	1.50 ± 0.71	
Ascites			
No	133	1.75 ± 1.04	0.334
Yes	90	2.05 ± 1.09	
History of hepatic encephalopathy			
No	175	1.84 ± 1.02	0.486
Yes	28	2.14 ± 1.35	
Child-Pugh class			
Class A	97	1.75 ± 0.99	0.716
Class B	66	2.00 ± 1.27	
Class C	40	2.00 ± 0.94	
EV grading			
Grade 1	81	1.85 ± 1.27	0.616
Grade 2	81	1.75 ± 0.85	
Grade 3	37	2.11 ± 1.05	
Grade 4	4	3.00 ± 0.95	
EV red color sign (RC)			
RC0 (none)	122	1.93 ± 1.17	0.912
HCS (hematocystic spots)	20	1.60 ± 0.55	
CRS (cherry red spots)	12	1.67 ± 1.16	
RWM (red wale markings)	49	1.92 ± 0.99	
EV form (F)			
F1	113	1.82 ± 1.19	0.780
F2	60	1.87 ± 0.83	
F3	30	2.14 ± 1.07	
EV risk signs			
No	131	1.88 ± 1.16	0.965
Yes	72	1.89 ± 0.90	
EV location (L)			
LI (inferior)	121	1.77 ± 0.94	0.046^∗^
LM (medialis)	69	1.82 ± 1.13	
LS (superior)	13	3.33 ± 1.16	
EV color (C)			
Cw (white varices)	142	1.89 ± 1.13	0.954
Cb (blue varices)	61	1.87 ± 0.92	

^∗^Statistically significant at *P* < 0.05. EV: esophageal varices; GAVE: gastric antral vascular ectasia.

**Table 5:** Correlation between portal hypertensive gastropathy score and different variables.

Parameter	Portal hypertensive gastropathy score (*n* = 203)	
	*R*	*P* value
Age (years)	0.187	0.194
Hemoglobin (g/dL)	0.325	0.021^∗^
Platelets (×10^9^/L)	−0.127	0.378
WBCs (×10^9^/L)	0.114	0.431
AST (U/L)	−0.066	0.651
ALT (U/L)	−0.014	0.922
Serum albumin (g/dL)	−0.072	0.620
Total serum bilirubin (mg/dL)	−0.080	0.581
International normalized ratio (INR)	−0.137	0.342
Creatinine (mg/dL)	0.086	0.555
Child-Pugh score	−0.228	0.111
Blood ammonia (*μ*g/dL)	0.295	0.037^∗^
Portal vein diameter (mm)	−0.142	0.325
Splenic longitudinal diameter (cm)	−0.383	0.006^∗^
Splenic vein diameter (mm)	−0.233	0.104

^∗^Statistically significant at *P* < 0.05. WBCs: white blood cells; ALT: alanine aminotransferase; AST: aspartate aminotransferase.

## Data Availability

The authors' institution does not allow public data access.
